# Photon-counting CT characterization of carotid perivascular adipose tissue: a layer-by-layer quantitative analysis. A preliminary analysis in an asymptomatic population

**DOI:** 10.1007/s00330-026-12481-z

**Published:** 2026-04-07

**Authors:** Luca Saba, Hatem Alkadhi, Erica Maffei, Roberta Sciciolone, Mahmud Mossa-Basha, Lorenzo Mannelli, Gennaro D’Anna, Antonella Balestrieri, Jasjit S. Suri, Filippo Cademartiri

**Affiliations:** 1 Department of Radiology, University of Cagliari, Cagliari, Italy; 2 Diagnostic and Interventional Radiology, University Hospital Zurich, University of Zurich, Zurich, Switzerland; 3 IRCCS SYNLAB SDN, Naples, Italy; 4 Department of Radiology, University of Washington, Seattle, WA USA; 5 Stroke Monitoring and Diagnostic Division, AtheroPoint™, Roseville, CA USA; 6 Department of Electrical and Computer Engineering, Idaho State University, Pocatello, ID USA; 7 Department of Computer Engineering, Graphic Era Deemed to be University, Dehradun, India; 8 University Centre for Research & Development, Chandigarh University, Mohali, India

**Keywords:** Computed tomography angiography, Photon-counting computed tomography, Carotid, Plaque, Perivascular adipose tissue

## Abstract

**Objective:**

Carotid atherosclerosis is a major contributor to ischemic stroke. While luminal stenosis has historically guided treatment decisions, growing evidence indicates that plaque composition, vascular inflammation and perivascular adipose tissue (PVAT) may be more closely linked to clinical outcomes and plaque vulnerability. This study aimed to characterize carotid PVAT using photon-counting computed tomography (PCCT) and to evaluate its spatial behavior and variability in a cohort of asymptomatic patients.

**Materials and methods:**

We retrospectively analyzed PCCT angiography data from 20 asymptomatic patients. A custom-developed Python algorithm was used to segment concentric perivascular layers from 1 mm to 5 mm around the carotid artery. For each layer, we quantified attenuation values in Hounsfield Units (HU) and voxel counts. Statistical comparisons were performed across layers and between sides.

**Results:**

Mean PVAT attenuation decreased progressively with increasing distance from the carotid wall. Significant differences were observed between inner and outer layers, particularly between the 1 mm and 3–5 mm annuli. Circle-by-circle analysis revealed substantial inter-individual variability in HU trends. Voxel count increased with annular thickness, but variability (SD and CV) also rose in outer layers. No significant differences were found between left and right carotid arteries in either attenuation or voxel distribution.

**Conclusion:**

Photon-counting CT enables detailed, layer-specific assessment of carotid PVAT. The observed attenuation patterns and inter-individual variability suggest that PVAT profiling may provide valuable insights into local vascular inflammation and plaque vulnerability. These findings support the potential of PCCT as a noninvasive tool for vascular risk stratification beyond luminal stenosis.

**Key Points:**

***Question*** *Can photon-counting CT enable a reliable, layer-by-layer quantitative characterization of carotid perivascular adipose tissue in asymptomatic patients beyond luminal stenosis assessment?*

***Findings**** Photon-counting CT demonstrated a progressive decrease in PVAT attenuation with increasing distance from the carotid wall and marked inter-individual variability across concentric layers*.

***Clinical relevance**** Layer-specific PVAT profiling with photon-counting CT may provide a noninvasive imaging marker of local vascular inflammation, supporting improved carotid risk stratification beyond stenosis severity, even in asymptomatic individuals*.

**Graphical Abstract:**

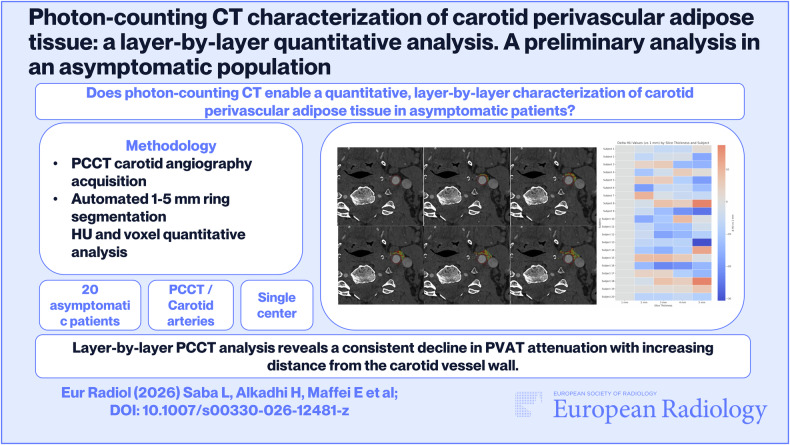

## Introduction

Stroke remains one of the leading causes of morbidity and mortality worldwide [1], with a significant proportion of cases attributable to carotid atherosclerotic disease. Since the 1980s, the degree of stenosis has been the primary parameter guiding therapeutic decisions. However, in recent years, growing evidence has demonstrated that carotid plaque characteristics, rather than stenosis severity alone, are more closely associated with the risk of cerebrovascular events [2, 3].

Beginning in the early 2000s, advancements in CT and MRI technology have facilitated the identification of several plaque vulnerability features [4] that were previously documented only through histological analysis, such as the presence of intraplaque hemorrhage (IPH) and fibrous cap rupture [5, 6]. Plaque vulnerability refers to the biological propensity of an atherosclerotic lesion to rupture or embolize, leading to an increased risk of transient ischemic attack or ischemic stroke, regardless of the degree of luminal narrowing [7]. Nonetheless, one of the most critical markers of plaque instability, according to histological data—namely, the inflammatory status—remains challenging to assess through conventional CT and MRI techniques. Currently, inflammation can be evaluated in vivo only through PET imaging or MRI techniques employing ultrasmall superparamagnetic iron oxide particles.

Insights from coronary artery CT studies have revealed that pericoronary adipose tissue, specifically its increased attenuation, serves as a sensitive marker of plaque inflammation and is strongly predictive of adverse cardiac outcomes [8]. Building on these findings, the characterization of pericarotid adipose tissue has recently emerged as an area of interest, with preliminary results showing promise [9, 10].

In 2021, photon-counting computed tomography (PCCT) was introduced into clinical practice. This novel technology offers a substantial improvement in spatial resolution, photon signal differentiation and tissue characterization capabilities, thereby also enhancing contrast resolution. In the present study, we aimed to assess the characteristics of carotid perivascular adipose tissue (PVAT) in a population of patients undergoing imaging of the supra-aortic trunks.

## Materials and methods

### Study design and patient population

The study was conducted in accordance with the principles outlined in the Declaration of Helsinki and received approval from the review board. Informed consent was waived due to the retrospective nature of the study. Patients who underwent PCCT angiography of the head and neck and all CT scans were performed in asymptomatic patients for clinical assessment of carotid atherosclerotic disease. According to the institutional standardized protocol, computed tomography angiography (CTA) of the carotid arteries is performed under the following conditions: (1) Carotid ultrasound findings indicated stenosis greater than 50%, as defined by the North American Symptomatic Carotid Endarterectomy Trial (NASCET) criteria. Ultrasound velocity measurements (PSV) were converted into NASCET-equivalent stenosis thresholds [11]. CTA was also performed if sonography revealed features suggestive of plaque vulnerability, such as ulcerations or an irregular surface. (2) Carotid ultrasound was inconclusive in assessing the degree of stenosis or plaque characteristics due to anatomical limitations.

### PCCT technique

All CT scans were conducted on a clinical PCCT (Naeotom Alpha, software version Syngo CT VA40, Siemens Healthineers) using a standard clinical scan protocol. All CT examinations were performed with the following acquisition parameters: the tube voltage was 120 kVp with automatic tube current modulation (IQ level 145, average effective current time product of 86.26 ± 13.39 mAs). The pitch was set at 0.8 with a rotation time of 0.25 s. The total collimation was 57.6 mm with a single collimation of 0.4 mm. The matrix was 1024 × 1024, and the field of view (FOV) was adjusted for each patient to optimally image the vessels from the aortic arch to the vertex. Contrast agent (50 mL, IOMERON® 400, Bracco) was injected into a peripheral vein of the arm using a coupled automated injector system with a flow of 4 mL/s followed by normal saline chaser (40 mL). Image acquisition was started with a delay of 8 s after reaching a threshold of 100 HU in an ROI placed in the ascending aorta.

A manufacturer-specific workstation (Syngo Via, VB60 version, Siemens Healthineers) was used to apply the reconstruction algorithm. In ultra-high-resolution (UHR) mode, the reconstructed in-plane spatial resolution was approximately 0.225 mm, with an isotropic voxel size achieved through resampling. The slice thickness was 0.4 mm, and the slice increment was 0.2 mm. The quantum iterative reconstruction level Q2 was used for all images.

### PVAT identification and analysis

To identify and quantify PVAT, we employed a custom-developed algorithm in Python created in our lab. The algorithm was used to manually trace the outer contour of the carotid plaque (specifically the slice corresponding to the maximum plaque thickness) on axial images, and no automatic approach was applied. Based on this contour, the algorithm automatically generated a concentric annulus with an operator-defined thickness, starting from 1 mm. The inclusion threshold for voxel attenuation was also operator-selectable, with the standard range set between −190 and −30 Hounsfield units (HU). Tracings were performed on native PCCT high-resolution reconstructions (UHR), which were resampled to isotropic voxels prior to annulus generation in a 1024 × 1024 matrix. The manually drawn contour was processed using a smoothing spline to ensure continuity and geometric stability before radial expansion. Concentric rings were generated through sequential morphological dilation of the initial contour, and only voxels within the adipose-compatible HU range were retained to minimize partial-volume contamination from the adventitia or plaque.

**Fig. 1:**
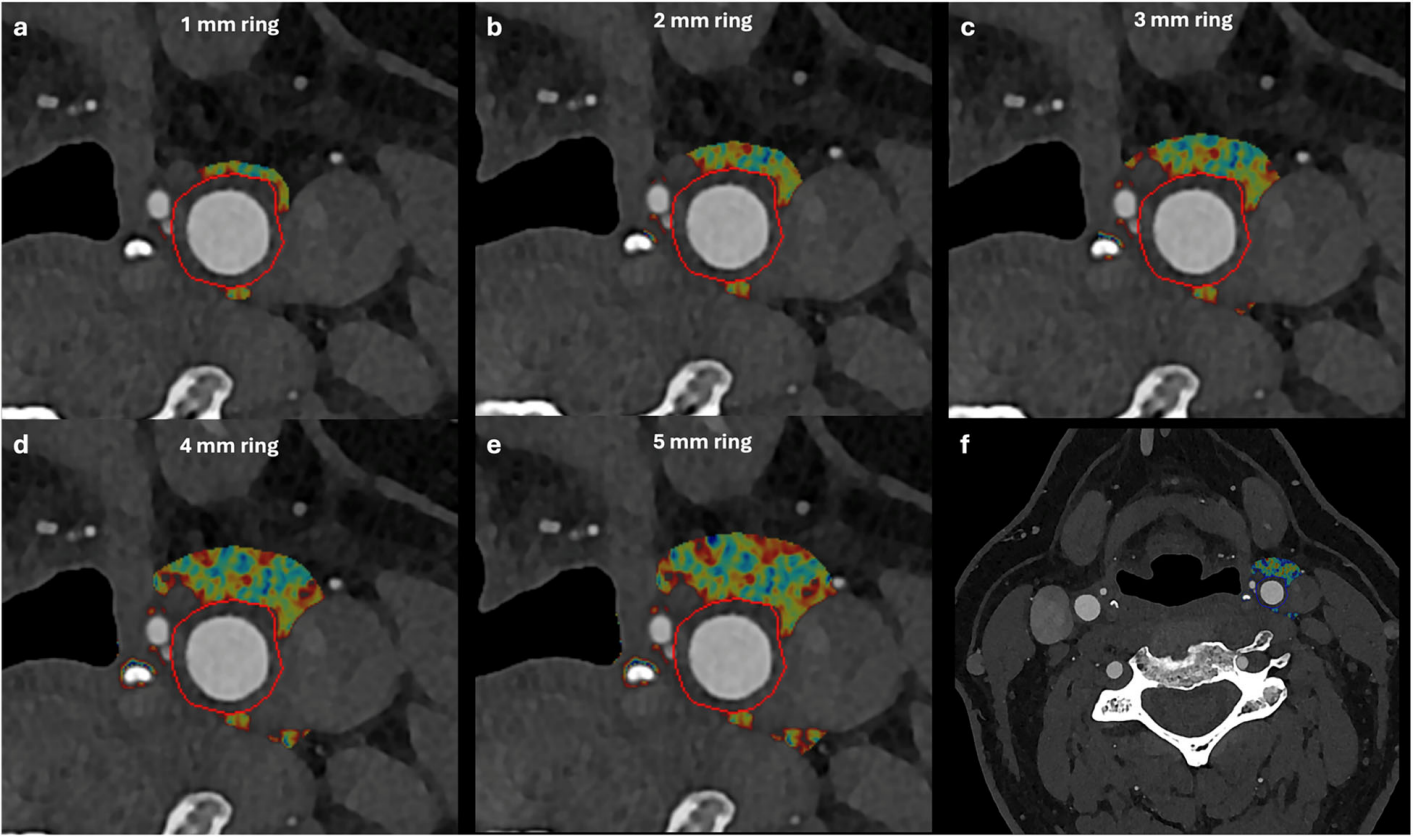
PVAT segmentation using concentric annular rings from the carotid wall. Axial cross-sections of the right carotid artery demonstrate the sequential expansion of perivascular concentric layers, ranging from 1 mm to 5 mm (**a**–**e**). Each image shows a jet color heatmap superimposed on the segmented PVAT, where attenuation values are visualized in a color gradient. The red circular contour marks the vessel wall, and the colored halo depicts the voxel-wise HU distribution. **f** The entire PCCT matrix

The algorithm then automatically computed the mean attenuation (HU) of the defined perivascular layer, reporting the number of voxels included within the selected region. Measurements were repeated using annular thicknesses of 1, 2, 3, 4, and 5 mm. This yielded overall PVAT values for each thickness. Additionally, to assess the behavior of PVAT at increasing distances from the carotid artery, the contribution of each individual 1-mm annular layer was separately calculated. This parameter was defined as the ‘circle’ value, allowing for a layer-by-layer analysis of PVAT attenuation (Fig. 1).

### Statistical analysis

Normality was assessed using the Shapiro–Wilk test. Since most continuous variables followed a normal distribution, parametric tests were applied. All quantitative data are reported as mean ± standard deviation (SD). HU values were compared across five different PCCT-derived perivascular annular thicknesses (1 mm, 2 mm, 3 mm, 4 mm, and 5 mm). Pairwise comparisons were performed using the paired Student’s *t*-test between thickness levels. These comparisons were conducted on both the overall mean HU values and the Circle HU values (defined as ROI-based measurements for each individual 1-mm annular layer). To adjust for multiple comparisons, the Benjamini–Hochberg false discovery rate (FDR) correction was applied. Analyses were performed both globally (regardless of laterality) and stratified by side (right vs left). In addition to HU metrics, the voxel count associated with each concentric layer (Circle) was analyzed. The distribution of voxel count was examined across the five thicknesses, and descriptive statistics were generated, including standard deviation, variance, and coefficient of variation (CV). The voxel count variability was visualized through bar and line plots, illustrating how variance and CV changed with increasing distance from the carotid wall. A heatmap analysis was also performed to evaluate the subject-wise ΔHU relative to the 1 mm reference layer, and radar charts were generated to visually summarize HU profiles across all thickness levels for each subject. All statistical analyses and visualizations were conducted using Python (version 3.11.8), employing dedicated scientific computing and plotting libraries. A *p*-value < 0.05 was considered statistically significant.

## Results

### Demographic data

A total of 20 patients (11 males and 9 females) were included in the study, with a mean age of 71.3 years and a standard deviation of 11.49 years. All patients analyzed were asymptomatic. The carotid arteries analyzed showed a mean stenosis of 24% (± 14%), measured according to the NASCET criteria.

### PVAT mean HU

**Fig. 2:**
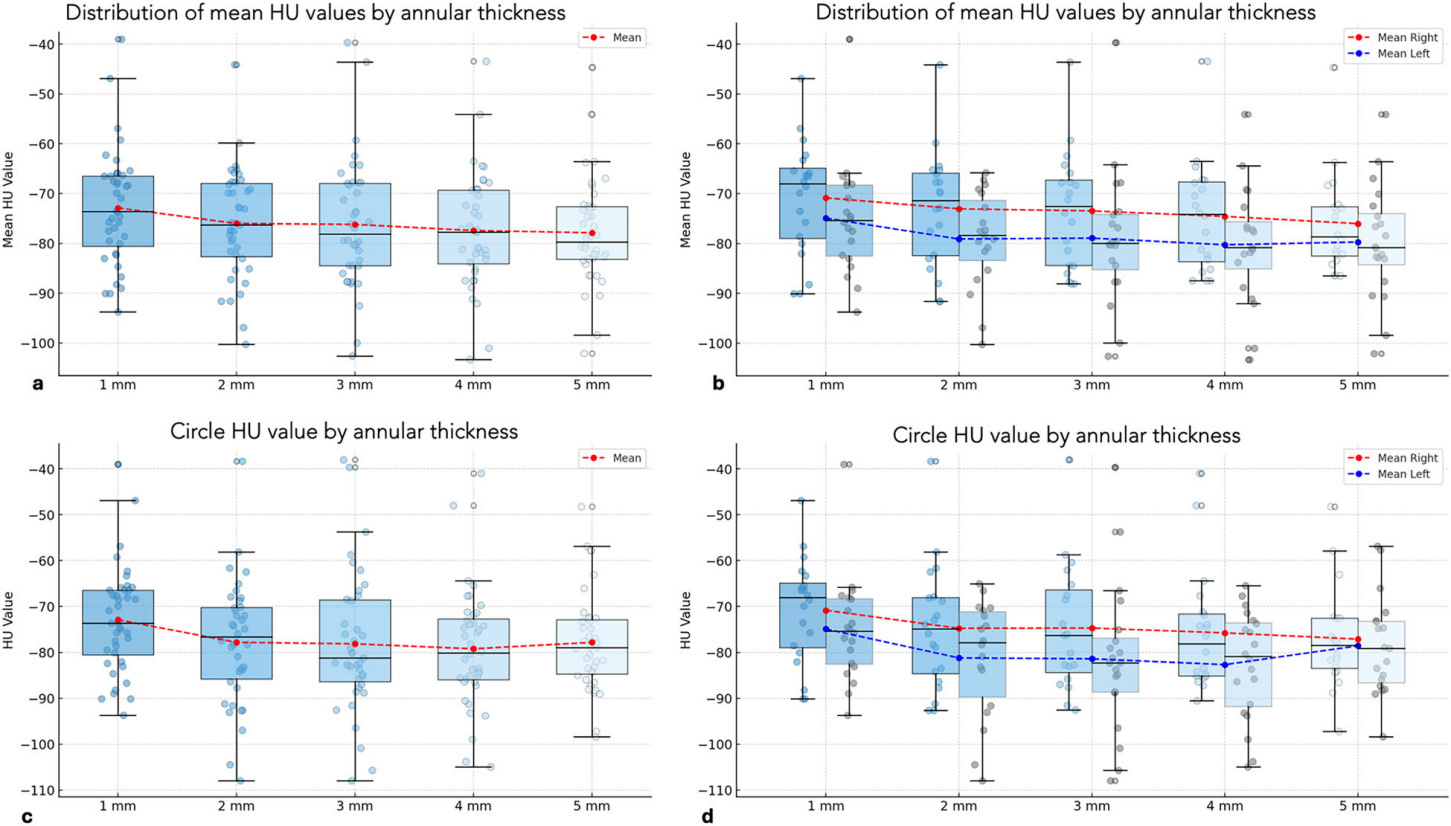
PVAT mean HU and Circle HU values. **a** Boxplot representation of the overall PVAT mean attenuation values across five concentric annular thicknesses (1–5 mm). The red dashed line represents the mean value at each layer. **b** Comparison of mean PVAT attenuation values stratified by side (right vs left). **c** Boxplot distribution of Circle HU values, defined as the mean HU within each discrete 1-mm annular layer. **d** Comparison of Circle HU values by side

Mean PVAT attenuation values, averaged over the entire segmented perivascular area at each of the five concentric thicknesses (1–5 mm), showed a progressive decrease in mean HU with increasing annular thickness. Pairwise comparisons revealed statistically significant differences between several thickness levels, particularly between the 1 mm and subsequent layers. After FDR correction, significance was retained for the majority of comparisons, especially involving the 1 mm reference layer (Table 1). The distribution and central tendency of these values are shown in Fig. 2a, where a gradual downward trend in HU values is visible.

**Table 1:** Pairwise comparisons of mean PVAT attenuation (HU) between concentric annular thicknesses from 1 mm to 5 mm

Comparison	Mean HU ± SD	t-statistic	Raw *p*-value	Corrected *p*-value (FDR)
1 mm vs 2 mm	−73.87 ± 10.45 vs −76.00 ± 11.06	3.241	0.0023	0.0056
1 mm vs 3 mm	−73.87 ± 10.45 vs −77.13 ± 11.45	3.933	0.0003	0.0011
1 mm vs 4 mm	−73.87 ± 10.45 vs −78.02 ± 11.09	4.647	0.0001	0.0002
1 mm vs 5 mm	−73.87 ± 10.45 vs −78.47 ± 10.14	4.790	0.0001	0.0002
2 mm vs 3 mm	−76.00 ± 11.06 vs −77.13 ± 11.45	2.597	0.0132	0.0166
2 mm vs 4 mm	−76.00 ± 11.06 vs −78.02 ± 11.09	3.197	0.0027	0.0056
2 mm vs 5 mm	−76.00 ± 11.06 vs −78.47 ± 10.14	2.992	0.0048	0.0081
3 mm vs 4 mm	−77.13 ± 11.45 vs −78.02 ± 11.09	2.662	0.0113	0.0162
3 mm vs 5 mm	−77.13 ± 11.45 vs −78.47 ± 10.14	2.074	0.04486	0.0498
4 mm vs 5 mm	−78.02 ± 11.09 vs −78.47 ± 10.14	1.093	0.2812	0.2811

Values represent mean HU ± SD for each pair of layers, along with the corresponding t-statistic, raw *p*-value, and FDR-corrected *p*-value

### Circle HU

**Fig. 3:**
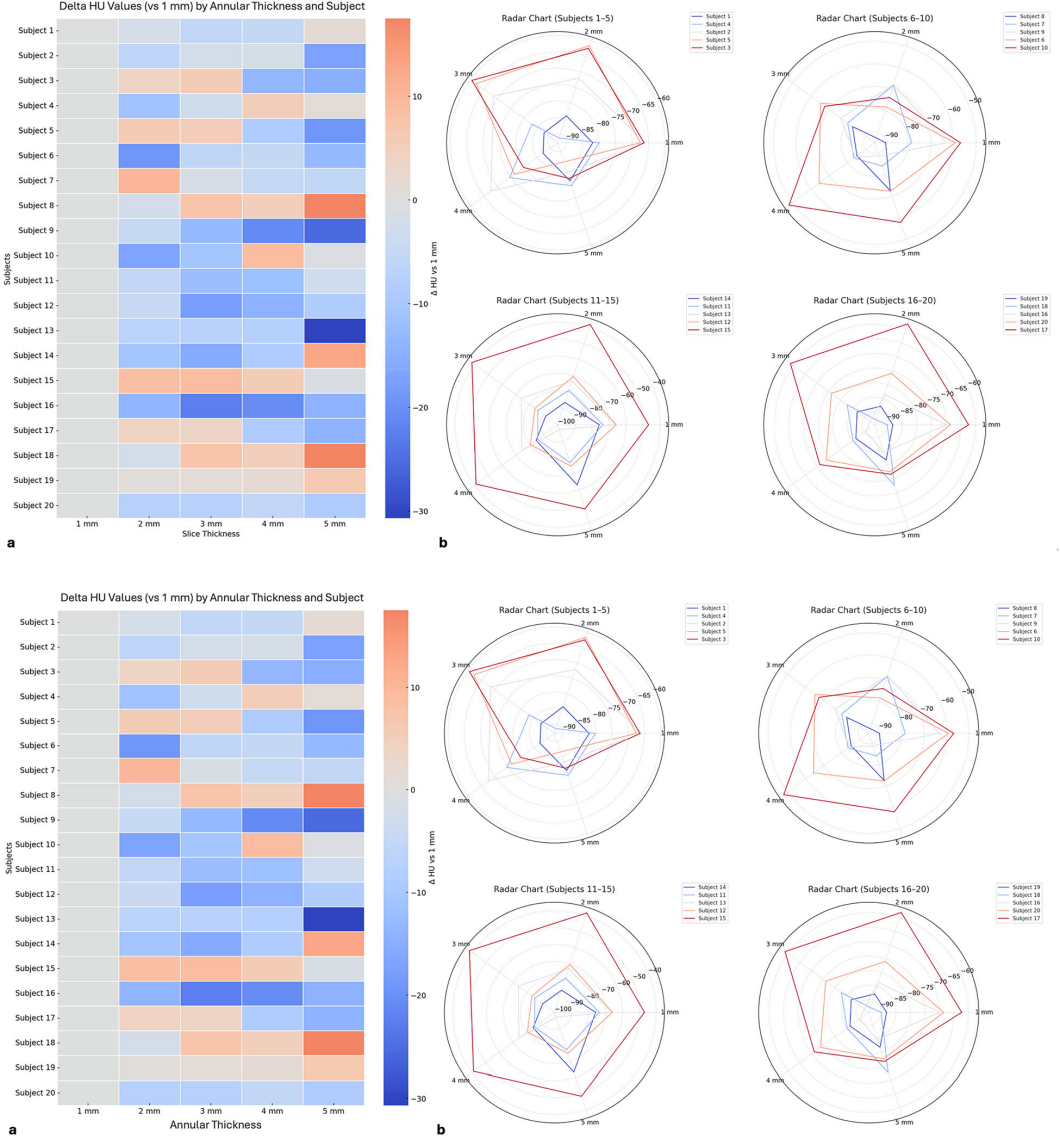
Subject-wise variability in Circle HU values. **a** Heatmap representing the ΔHU between each concentric layer (2–5 mm) and the 1 mm reference layer for each subject. **b** Radar plots showing HU values across the five layers for four subsets of subjects (1–5, 6–10, 11–15, 16–20). Distinct attenuation profiles emerge, with some subjects exhibiting linear trends and others showing nonlinear or inverted behavior

When analyzing Circle HU values, defined as layer-specific mean HU values from discrete annular ROIs of 1 mm thickness, the attenuation followed a similar decreasing trend with increasing distance from the vessel wall. The 1 mm layer consistently showed higher attenuation values than the outer layers. Statistical analysis confirmed significant differences across thickness levels, particularly between the 1 mm layer and the 3–5 mm circles (Table 2). A subject-wise heatmap of ΔHU relative to the 1 mm layer (Fig. 3a), along with radar plots (Fig. 3b), demonstrates substantial inter-individual variability in HU profiles, with some subjects showing nonlinear or inverted trends across layers.

**Table 2:** Pairwise comparisons of Circle HU values, defined as the layer-specific mean attenuation (HU) obtained from the discrete 1-mm annular ROIs

Comparison	Mean HU ± SD	t-statistic	Raw *p*-value	Corrected *p*-value (FDR)
1 mm vs 2 mm	−73.87 ± 10.45 vs −78.06 ± 13.50	3.069	0.0040	0.0135
1 mm vs 3 mm	−73.87 ± 10.45 vs −78.89 ± 14.18	3.259	0.0024	0.0122
1 mm vs 4 mm	−73.87 ± 10.45 vs −79.47 ± 13.17	3.569	0.0010	0.0103
1 mm vs 5 mm	−73.87 ± 10.45 vs −77.79 ± 11.06	2.029	0.0499	0.1247
2 mm vs 3 mm	−78.06 ± 13.50 vs −78.89 ± 14.18	0.662	0.5119	0.7019
2 mm vs 4 mm	−78.06 ± 13.50 vs −79.47 ± 13.17	0.783	0.4387	0.7013
2 mm vs 5 mm	−78.06 ± 13.50 vs −77.79 ± 11.06	−0.118	0.9067	0.9067
3 mm vs 4 mm	−78.89 ± 14.18 vs −79.47 ± 13.17	0.483	0.6317	0.7054
3 mm vs 5 mm	−78.89 ± 14.18 vs −77.79 ± 11.06	−0.567	0.5739	0.7021
4 mm vs 5 mm	−79.47 ± 13.17 vs −77.79 ± 11.06	−1.124	0.2685	0.5371

The table reports mean HU ± SD for each layer comparison, together with t-statistics, raw *p*-values, and FDR-corrected *p*-values

### Circle number of voxels

**Fig. 4:**
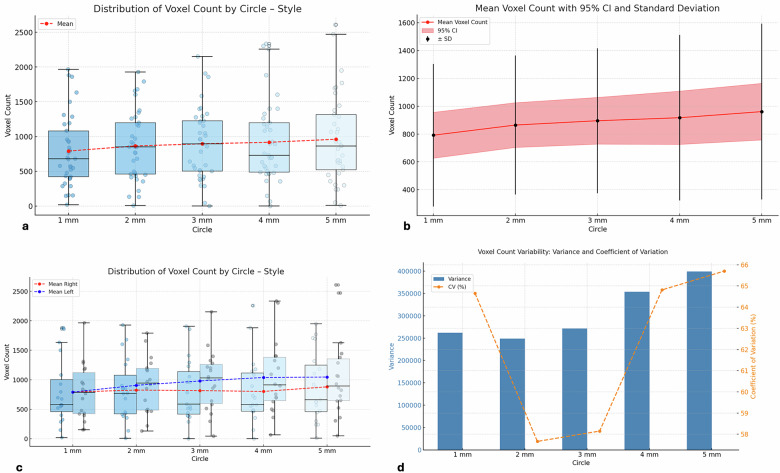
Voxel count analysis across circles. **a** Boxplot of voxel count distribution for each concentric circle (1–5 mm). **b** Line plot of the mean voxel count with superimposed 95% confidence intervals and standard deviation. **c** Comparison of voxel count distribution between right and left sides. **d** Combined bar and line plot showing the variance (blue bars) and coefficient of variation (orange dashed line) of voxel counts for each circle. Both metrics increase with concentric thickness, suggesting growing heterogeneity in segmentation

The number of voxels per annular circle increased with concentric thickness, reflecting the increasing volume encompassed by the segmentation. The average voxel count rose steadily from Circle 1 mm to Circle 5 mm (mean range: ~900 to ~1100 voxels), but this was accompanied by increasing variability at greater thicknesses. A graphical exploration of voxel count distribution (Fig. 4a) demonstrated wider dispersion in the 4 mm and 5 mm circles. The confidence bands in Fig. 4b highlight the relative overlap in earlier layers and separation beyond the 3 mm layer, reinforcing the notion that segmentation becomes more inconsistent at greater distances.

Further statistical exploration (Fig. 4d) revealed an increasing pattern in both variance and coefficient of variation (CV%) across the five circles. This suggests not only a higher spread in absolute terms, but also a higher relative variability, possibly reflecting anatomical irregularities or segmentation effects in peripheral layers. Full summary metrics are available in the Table 3.

**Table 3:** Pairwise statistical comparisons of voxel counts across concentric annular circles (1 mm to 5 mm)

Comparison	t-statistic	*p*-value	FDR-corrected *p*-value
1 mm vs 2 mm	−1.608	0.1164	0.2912
1 mm vs 3 mm	−1.825	0.0762	0.2544
1 mm vs 4 mm	−1.856	0.0715	0.2541
1 mm vs 5 mm	−2.305	0.0270	0.2012
2 mm vs 3 mm	−0.999	0.3240	0.3891
2 mm vs 4 mm	−1.026	0.3113	0.3889
2 mm vs 5 mm	−1.329	0.1920	0.3840
3 mm vs 4 mm	−0.529	0.6003	0.6003
3 mm vs 5 mm	−1.007	0.3205	0.3891
4 mm vs 5 mm	−0.946	0.3502	0.3892

The table presents t-statistics, raw *p*-values, and FDR-corrected *p*-values for all circle-to-circle voxel count comparisons

### The effect of sidedness

When stratifying all analyses by laterality (right vs left carotid artery), no systematic difference in attenuation or voxel count was observed across the five concentric layers (Fig. 2b). Paired *t*-tests comparing corresponding circles on the left and right side did not reveal significant differences after FDR correction. In both Circle HU and voxel count distributions (Figs. 2d, 4c), the trends for right and left sides were nearly superimposable, with only minimal divergence. Mean HU values showed slightly lower values on the left in outer circles (4–5 mm), but these differences were not statistically significant.

## Discussion

This study provides a detailed characterization of carotid PVAT using photon-counting computed tomography, an emerging imaging modality offering superior spatial and contrast resolution compared to conventional CT [12]. Currently, there are no studies in the literature evaluating this type of parameter on PCCT, representing a novel approach considering the modality’s increased spatial resolution, matrix density, and contrast resolution.

Our findings demonstrate a consistent and progressive decrease in PVAT attenuation with increasing distance from the carotid wall, as well as significant inter-individual variability in the distribution and profile of PVAT HU values. These observations are in line with previous studies conducted in the coronary circulation, where perivascular fat attenuation has been validated as a surrogate marker of vascular inflammation and a predictor of adverse cardiovascular events. In particular, it has been demonstrated in the coronary artery that Hounsfield Units (HU) attenuation decreases progressively with increasing distance from the vessel wall [13]. In our specific case, the patients included were all asymptomatic, and while this supports the presence of a detectable inflammatory signal even in subclinical carotid atherosclerosis, it does not allow us to infer plaque stability, as no longitudinal or outcome data are available.

The ability of PCCT to quantify subtle changes in PVAT attenuation across millimetric concentric layers may represent a major step forward in the noninvasive assessment of local vascular inflammation, particularly when compared with conventional high-resolution EID-CT. Unlike EID detectors, which are limited by light scatter, electronic noise, and edge-blur effects that can obscure fine perivascular gradients, PCCT provides true high-resolution imaging with markedly reduced halo artifacts and an in-plane resolution of 0.225 mm in UHR mode. These technical advantages allow PCCT to capture attenuation differences that would be partially or completely masked on EID-CT systems. The significant differences in attenuation between the innermost 1 mm layer and outer circles support the concept that the PVAT closest to the vessel wall may be more directly influenced by inflammatory signals originating from the vascular wall itself, a phenomenon previously described as the “outside-in” paradigm of atherosclerotic inflammation [14].

Importantly, the heatmap and radar chart visualizations revealed that not all patients follow the same attenuation pattern across layers, with some showing nonlinear or even inverted trends. This inter-individual heterogeneity suggests that PVAT behavior in asymptomatic subjects may be influenced by a range of anatomical, metabolic, or pathophysiological factors that warrant further investigation. It is interesting to observe that some individuals exhibit significantly higher PVAT values compared to others.

**Fig. 5:**
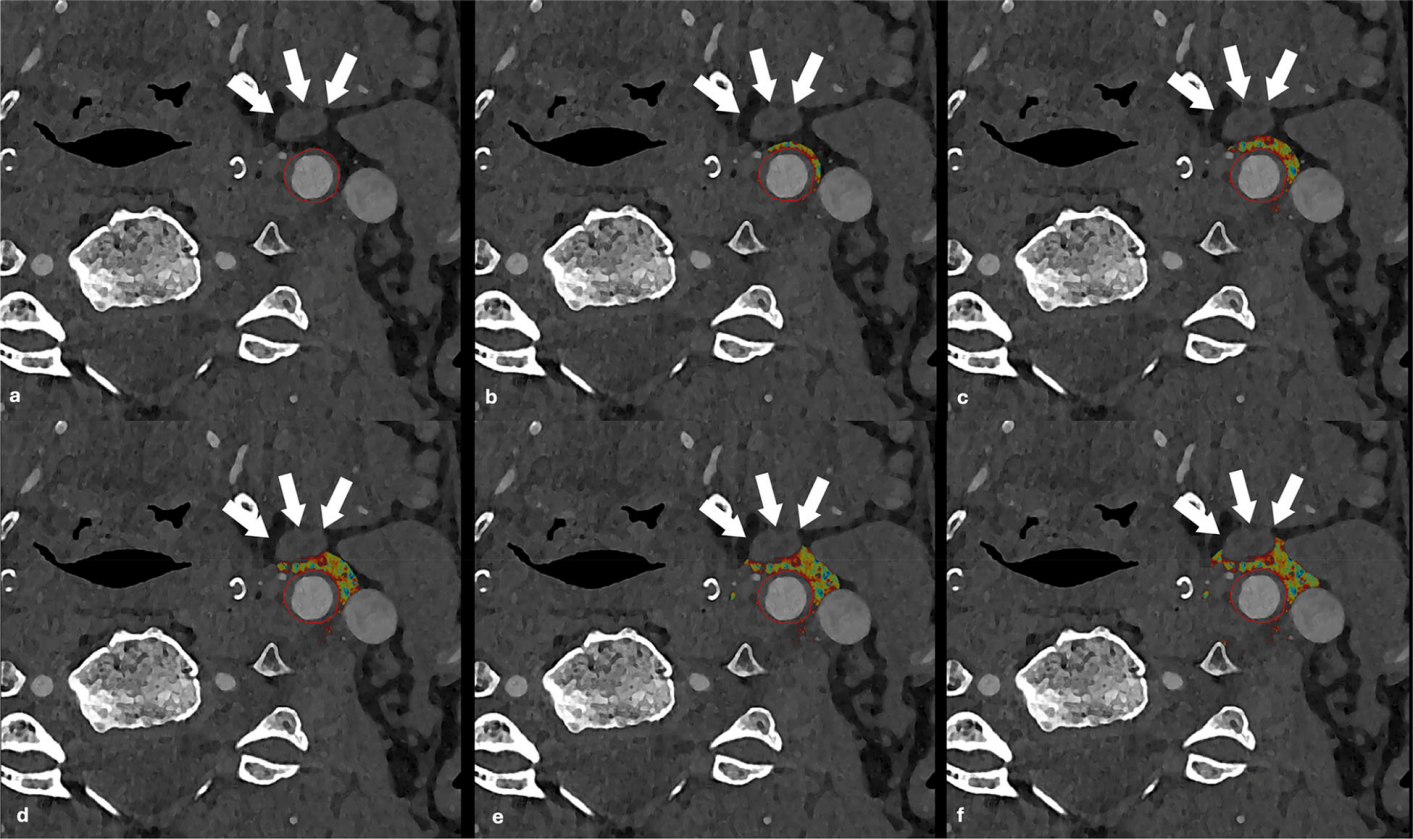
Example of PVAT segmentation with adjacent lymph node. Axial cross-sections illustrate how a lymph node (indicated by white arrows) adjacent to the carotid artery can interfere with perivascular segmentation, particularly at larger concentric thicknesses. In the panel (**a**), the CTA axial scan with the outer border of the plaque traced. As the segmented layers increase from 1 mm to 5 mm (**b**–**f**), the inclusion of non-adipose tissue, specifically the lymph node, results in visible distortion of the perivascular region. The jet heatmap demonstrates heterogeneous attenuation patterns that differ from the typical PVAT signature, especially in outer circles (**d**–**f**). This highlights how anatomical complexity can influence voxel composition and impact on quantitative measurements of attenuation and volume

This not only emphasizes the importance of optimizing segmentation strategies to isolate true perivascular fat, particularly when analyzing broader regions, but also highlights that the landscape of PVAT in carotid arteries is fundamentally different from that of the coronary arteries. Indeed, coronary arteries are physiologically surrounded by fat, with minimal interference from other anatomical structures. In contrast, the pericarotid space often contains additional elements such as lymph nodes (Fig. 5) or small vascular and neural structures. In our model, particular attention was devoted to ensuring that all non-adipose components were excluded. This was reliably achieved through the use of strict HU-based thresholds (−190 to −30 HU), which allowed the algorithm to retain only true adipose tissue and systematically discard lymph nodes, vascular structures, and other non-fat elements. Even when these are excluded from segmentation, their proximity may still influence local fat attenuation. For example, a nearby lymph node could alter the attenuation characteristics of the surrounding adipose tissue due to local inflammatory activity, potentially confounding PVAT analysis. Notably, no significant differences were observed between the right and left carotid arteries, suggesting that PVAT attenuation is symmetric in the absence of side-specific disease or asymmetry in anatomical structures. However, the small sample size and the asymptomatic nature of the population may have limited the detection of lateralized patterns, and although no statistically significant differences were observed, the left carotid consistently showed nominally lower attenuation values and smaller PVAT volumes compared with the right side.

Our study has a number of strengths. It introduces and validates a reproducible, semi-automated method for PVAT segmentation and analysis using PCCT. Moreover, by analyzing both overall and layer-specific values (Circle HU), we provide a detailed insight into the spatial behavior of perivascular fat.

Nevertheless, some limitations must be acknowledged. First, the study population was relatively small and included only asymptomatic patients, and the very high variation observed among the 20 subjects may partly reflect this limited sample size. As a consequence, the generalizability of our findings to symptomatic or high-risk populations may be reduced. Second, the segmentation was based on anatomical proximity rather than histological validation, and although the HU thresholds used are standard for adipose tissue, some contamination by adjacent tissues cannot be excluded, particularly in the outer layers. Third, longitudinal clinical outcomes were not available, precluding correlations between PVAT features and cerebrovascular events, which could be evaluated in future studies.

## Abbreviations

CTA: Computed tomography angiography
CV: Coefficient of variation
HU: Hounsfield unit
PCCT: Photon-counting CT
SD: Standard deviation
